# Central Asia Cold Case: Siberian Pine Fingers New Suspects in Growth Decline CA 1700 CE

**DOI:** 10.3390/plants14020287

**Published:** 2025-01-20

**Authors:** David M. Meko, Dina F. Zhirnova, Liliana V. Belokopytova, Yulia A. Kholdaenko, Elena A. Babushkina, Nariman B. Mapitov, Eugene A. Vaganov

**Affiliations:** 1 Laboratory of Tree-Ring Research, University of Arizona, 1215 E. Lowell Street, Tucson, AZ 85721-0045, USA; dmeko@arizona.edu; 2 Khakass Technical Institute, Siberian Federal University, 27 Schetinkina Street, Abakan 655017, Russia; dina-zhirnova@mail.ru (D.F.Z.); kropacheva_yulechka@mail.ru (Y.A.K.); babushkina70@mail.ru (E.A.B.); 3 Institute of Ecology and Geography, Siberian Federal University, 79 Svobodny Pr., Krasnoyarsk 660041, Russia; eavaganov@hotmail.com; 4 Department of Biology and Ecology, Toraighyrov University, 64 Lomov Street, Pavlodar 140008, Kazakhstan; mapitov@mail.ru; 5 Department of Dendroecology, V.N. Sukachev Institute of Forest, Siberian Branch of the Russian Academy of Science, 50/28 Akademgorodok, Krasnoyarsk 660036, Russia

**Keywords:** conifers, tree-ring width, severe growth suppression, abiotic factors, stress event

## Abstract

Tree-ring width chronologies of *Pinus sibirica* Du Tour from near the upper treeline in the Western Sayan, Southern Siberia are found to have an exceptional (below mean–3SD) multi-year drop near 1700 CE, highlighted by the seven narrowest-ring years in a 1524–2022 regional chronology occurring in the short span of one decade. Tree rings are sometimes applied to reconstruct seasonal air temperatures; therefore, it is important to identify other factors that may have contributed to the growth suppression. The spatiotemporal scope of the “nosedive” in tree growth is investigated with a large network of *P. sibirica* (14 sites) and *Larix sibirica* Ledeb. (61 sites) chronologies, as well as with existing climatic reconstructions, natural archives, documentary evidence (e.g., earthquake records), and climate maps based on 20th-century reanalysis data. We conclude that stress from low summer temperatures in the Little Ice Age was likely exacerbated by tree damage associated with weather extremes, including infamous Mongolian “dzuds”, over 1695–1704. A tropical volcanic eruption in 1695 is proposed as the root cause of these disturbances through atmospheric circulation changes, possibly an amplified Scandinavia Northern Hemisphere teleconnection pattern. Conifer tree rings and forest productivity recorded this event across all of Altai–Sayan region.

## 1. Introduction

Tree rings are a well-known and widespread proxy for seasonal climate reconstructions [1,2,3]. However, growth suppression or release in trees can also be caused by abrupt events—pest infestations, earthquakes, volcanic eruptions, landslides, floods, fires, etc. [4,5,6,7,8,9,10]. Such events may or may not be causally linked to anomalies of seasonal climate. For reliable climate reconstructions, we must distinguish as much as possible growth departures driven by the reconstructed variable from those due to other factors. Case studies of exceptional fluctuations in climate proxies through comparison with documentary evidence and other natural archives recording environmental conditions can pave the way in flagging possible inaccuracies in the climatic reconstructions.

Recently, we documented an exceptional decadal decline, or ‘nosedive’, in tree-ring width (TRW) and wood anatomical parameters of Siberian pine (*Pinus sibirica* Du Tour) throughout the Western Sayan highlands, beginning ca 1700 CE [11,12]. The finding spurred us to investigate whether the phenomenon was limited to *P. sibirica* and certain habitats, and whether a seasonal climate (temperature) anomaly or some other natural extreme or event was responsible. Tree-ring chronologies from continental temperate Asia have been widely used to reconstruct temperatures [1,13,14,15,16]. Some chronologies in these studies have an abrupt drop in growth around 1700, but the feature went unnoticed, not standing out against the background of other fluctuations, such as a long-term growth decrease during the 19th century (end of Little Ice Age) and the subsequent faster growth due to contemporary warming [1,13,15]. The near-1700 nosedive in *P. sibirica* growth is too sharp, however, to assume that it was driven by seasonal temperature fluctuation alone, so we hypothesized that there was some other stressful event that caused or enhanced this growth suppression.

This study explores possible alternative explanations for the near-1700 nosedive in *P. sibirica* growth. Growth fluctuations in *P. sibirica and* Siberian larch (*Larix sibirica* Ledeb.), another climate-sensitive and co-existing conifer frequently used for reconstructions [17], are studied in the context of natural factors and events that could plausibly have negatively impacted tree growth in the western Sayan Mountains near 1700 across the eastern part of Greater Central Asia.

## 2. Results

Since this phenomenon occurred several centuries ago, the lack of direct instrumental data made us investigate its possible causes through circumstantial evidence. This evidence is in the form of natural proxies recording environmental fluctuations (tree rings, lake sediments, glaciers’ ice cores etc.), climatic reconstructions based on those proxies, and historical chronicles. Here we assess the near-1700 CE anomaly in our chronologies of *P. sibirica* tree-ring width in the context of this available evidence.

### 2.1. Fingerprint of Event

**Figure 1:**
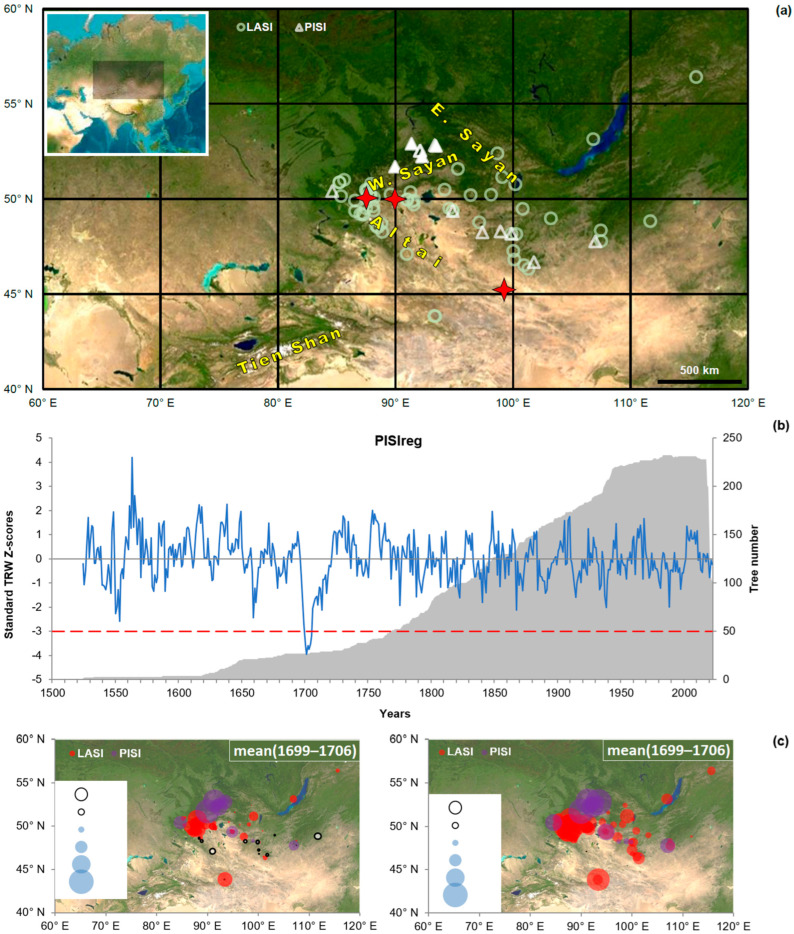
Growth suppression in tree-ring width of *Pinus sibirica* Du Tour (PISI) and *Larix sibirica* Ledeb. (LASI). (**a**) Map of the study area with marked locations of PISI (triangles) and LASI (circles) sampling sites, and epicenters of the strongest earthquakes (asterisks). Four filled triangles represent PISI chronologies with the most severe growth suppression used to develop regional chronology PISIreg. Insert map shows the location of the study area in Asia; (**b**) Regional chronology PISIreg, 1524–2022, as Z-scores. Red line represents the Z = −3.0 threshold of growth suppression; grey shading represents sample depth; (**c**) Geographical distribution of mean (left) and minimum (right) Z-score values for all 14 PISI and 61 LASI chronologies. Colored circles represent negative Z-scores for PISI (purple) and LASI (red); white circles represent positive Z-scores; circle diameters (see legend) represent absolute value.

**Figure A1:**
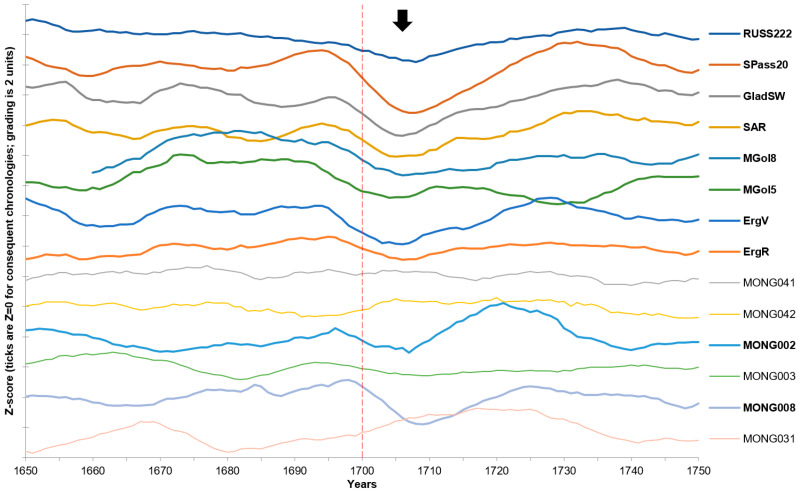
Standard chronologies of *Pinus sibirica* (1650–1750) smoothed by 11-year moving average. Chronologies are plotted in Z-score units and shifted sequentially by 2 units along vertical axis for clarity. The same relative y-scale applies to all plots, tick marks represent Z = 0 for each chronology. Vertical dashed line marks calendar year 1700; vertical arrow marks growth suppression; chronologies with synchronous growth suppression after 1698 are represented by thick lines and marked with bold labels.

The primary tree-ring data for our analysis consist of 14 pine (*P. sibirica*) and 61 larch (*L. sibirica*) chronologies with time coverage back to at least 1690 CE available in a study area (40–60° N 60–120° E), centered on the Western Sayan Mountains (Figure 1a; Table A1, Figure A1). We defined the “nosedive” as the sharp multi-year suppression of growth starting ca 1700 CE. The nosedive was evident in 10 of the pine and 19 of the larch chronologies, and most seriously expressed (lowest values of Z-scores) in four near-timberline Western Sayan *P. sibirica* chronologies, some of which were previously shown as sensitive to temperature [11], A regional chronology PISIreg, developed from these four sites and containing more than 25 individual tree series covering 1690, is therefore used here to characterize the ca-1700 nosedive. The seven lowest values of PISIreg in the 499 years of its coverage (1524–2022 CE) are the consecutive years 1699–1705, and in six of these seven years, the Z-score of PISIreg is below −3 (Figure 1b). A less pronounced growth suppression is also present in several *P. sibirica* chronologies from the Altai Mountains and the Mongolian Plateau.

**Figure 2:**
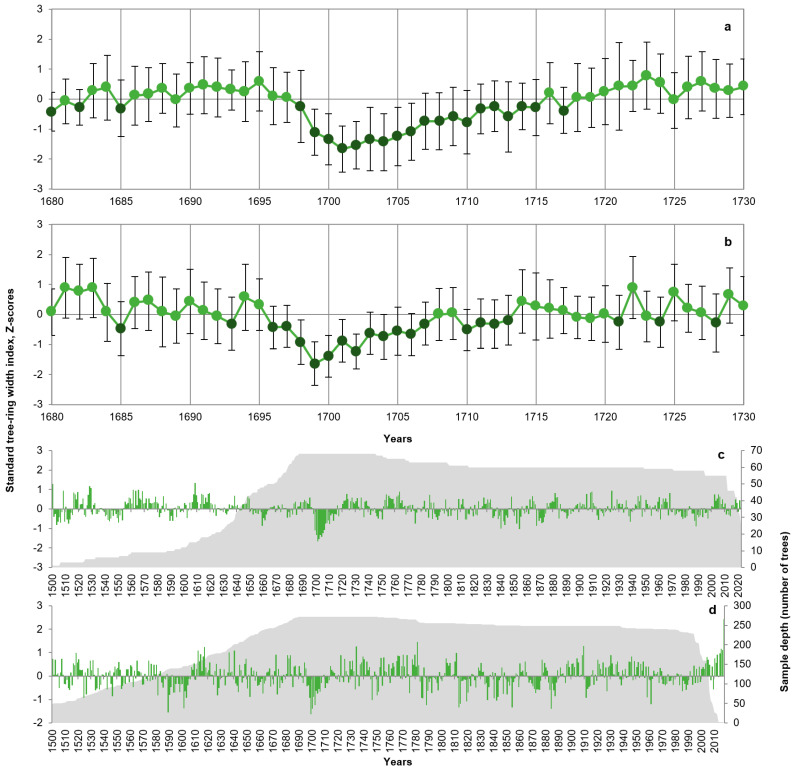
Growth suppression in tree-ring width of *Pinus sibirica* (**a**,**c**) and *Larix sibirica* (**b**,**d**) individual trees covering the period of the near-1700 CE nosedive. Trees were selected from growth-suppressed chronologies, and indices were transformed into Z-scores (standard series for 68 trees from 10 pine chronologies, 272 trees from 19 larch chronologies). (**a**,**b**) Mean values (markers) and SD (standard deviation; error whiskers) for 1680–1730; years with mean Z-scores below zero (mean + SE < 0; SE, standard error) are presented by darker shade of markers. (**c**,**d**) Time series of mean tree-ring index (Z-scores; bars) and sample depth (grey shading) for those trees since 1500.

The nosedive is characterized in pine and larch tree rings of individual trees (selected from chronologies where it occurred) as an abrupt initial drop in growth in 1698–1699 CE, followed by several years of very low growth and a gradual recovery toward normal growth (Figure 2a,b). The entire episode covered roughly 15 years in pine and 8 years in larch. Years with the largest departures were similar for pine (1699–1706) and larch (1699, 1700, 1702). For both species, growth suppression was accentuated at higher elevations. It is also the most expressed growth departure common for those trees since 1500, especially for pine; the only other deviation of similar magnitude and duration is the recent positive trend in larch growth (Figure 2c,d).

Suppression of growth in larch and pine chronologies in the study area during the nosedive window 1699–1706 is most pronounced in the Western Sayan Mountains (pine) and the eastern part of the Russian Altai Mountains (larch) (Figure 1c), as supported by other studies [7,11,17,18]. Mean of Z-scores below −2 and minimum values below −3 (extreme suppression) were observed only in one chronology outside that territory, but moderate suppression extended to China and Mongolia (Table A1).

### 2.2. Paleoclimatic and Tectonic Context

Summer temperature reconstructions in the study area, based on TRW [19,20], blue intensity [16] and maximum latewood density [15] of tree species other than *P. sibirica*, are significantly (*p* < 0.05) positively correlated with PISIreg both at high and low frequencies (Table 1). Correlations are higher for 1650–1750 than for the full overlap of records and generally increase with the smoothing of the time series. These reconstructions have negative z-scores for the nosedive window, 1699–1706, indicating cool summers at the time. Correlation with PISIreg is consistently higher for reconstructions from larch than from spruce (*Picea schrenkiana* Fisch. & C.A.Mey.). The temperature reconstructions reach maximum correlation with PISIreg at lag = 0 before smoothing and at high frequencies, indicating synchrony in annual departures, but at lags of several years at low frequencies (Figure 2).

Documentary records list neither volcanic eruptions nor any earthquakes in the study area at the time of the nosedive. Strong earthquakes (intensity of 10–11, Mercalli scale) were reported in recent history in the Altai Mountains in 1761 CE, 1957 CE and 2003 CE. Epicenters are marked in Figure 1a. Eight-year-growth departures of pine and larch, as Z-scores, after these earthquakes are much less severe than the PISIreg departure during the 1699–1706 nosedive (Table 2). Suppression of tree growth after these earthquakes was moreover observed in only a few chronologies.

### 2.3. Climate Teleconnections

**Figure 3:**
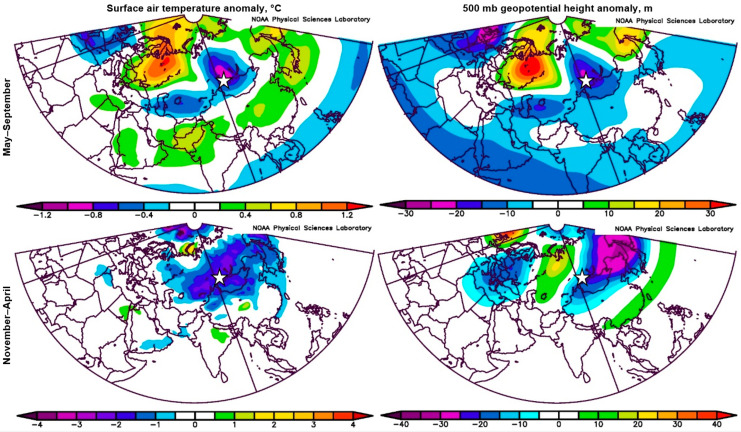
Composite anomaly maps of surface air temperature (**left**) and 500 mb geopotential height (**right**) in warm (May–September, **top**) and preceding cold (November–April, **bottom**) seasons for the years 1938 and 1988. These are the years with the lowest *P. sibirica* growth in the 20th century according to regional chronology PISIreg (white asterisk). Maps were developed by the web-based 20th Century Reanalysis V2 tool (https://psl.noaa.gov/cgi-bin/data/composites/plot20thc.v2.pl, accessed on 14 October 2024).

Only two 20th-century years, 1938 and 1988, had PISIreg z-scores lower than −2.0; these are the 14th and 15th lowest values of PDSIreg over the full 1524–2022 CE record, although not nearly as extreme as in the nosedive. The composite 500 mb height and 2 m air temperature anomaly patterns for those years highlight cyclonic flow and cold conditions in the study area in both seasons as signatures for extremely narrow *P. sibirica* rings in the Western Sayan (Figure 3). In the warm season, PISIreg is under anomalous northerly flow and is near the core of a well-defined central Asia cold anomaly. In the cool season, PISIreg is near the center of a broad zonally oriented core of anomalous cold stretching across southern Siberia and is directly under very strong anomalous northerly flow at 500 mb.

The anomalous 500 mb flow in the 1938/1988 composite is strongly meridional, with centers of action (highs or lows of anomalous heights) in the warm season resembling the positive mode of the “Eurasia 1”, or “Scandinavia” teleconnection circulation pattern [21]. This is one of 10 preferred modes of Northern Hemisphere circulation that can explain the simultaneous occurrence of abnormal weather patterns across vast distances (http://www.cpc.ncep.noaa.gov/data/teledoc/telecontents.shtml; accessed on 15 November 2024) and is known to significantly affect the study area [22]. The cool season 500 mb composite map has no clear resemblance to the Scandinavia pattern.

## 3. Discussion

### 3.1. Prime Suspect: Cold Spell

A decadal-scale exceptionally cold period is the natural suspect in the PISIreg nosedive. Energy limitation is after all the generally accepted foundation for temperature reconstruction from the ring width of boreal and subalpine trees [23,24]. Moreover, a long cold period could impact forest vitality and resistance to other stress factors [4,25]. Growth reduction in conifer forests in the study area in cold periods increases with elevation [26,27], which is consistent with amplified reduction in the subalpine *P. sibirica* chronologies of PISIreg. The nosedive occurred during the Little Ice Age, and the several decades before and after the nosedive are estimated to have been cold, possibly exacerbated by the Maunder solar activity minimum of 1645–1715 [19,28,29]. Summer temperature reconstructions and temperature-sensitive tree-ring chronologies from different species and tree-ring variables (TRW, wood density, and blue intensity) support 1699–1706 as a cold period in the study area [11,13,14,15,16,17,18,19,20]. Anomalies consistent with the nosedive were also observed in temperature-sensitive wood cell parameters of *P. sibirica* in Western Sayan [12]. It is likely that all growth processes were inhibited at the time—both cell division in the cambium and carbon accumulation during tracheid differentiation, since wood density is determined by cell structure [30,31].

The concentration of NH4^+^ and HCOO^−^ ions, originating from forest emissions, in the Belukha ice cores reached a historical minimum in the decade 1710–1720 [29]. A comprehensive temperature reconstruction based on Lake Teletskoe sediments (Altai), including bromine reflecting the state of the vegetation, also had a minimum in ~1710 [32]. These data indicate a regional decline in the productivity of the forest ecosystems soon after the investigated event on a decadal scale.

**Figure 4:**
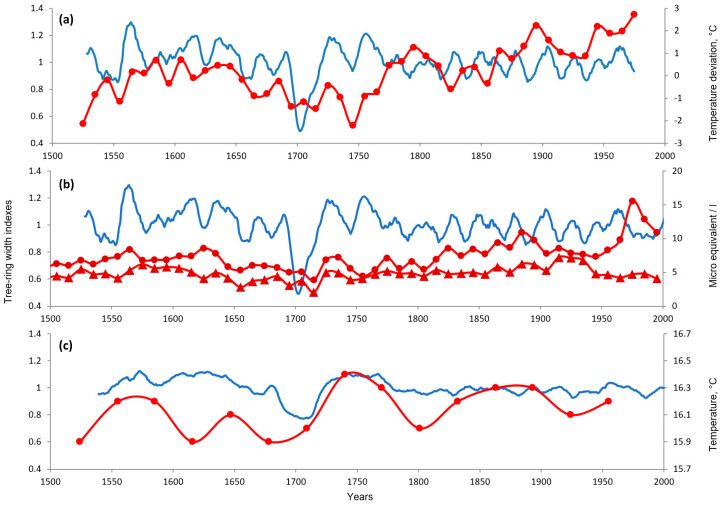
Growth suppression ca 1700 CE in PISIreg (blue line without markers) in the context of climate reconstructions and natural archives of decadal resolution (red lines with markers). (**a**) July temperature at the Hulun Lake (Mongolia) reconstructed from pollen in lake sediments (average resolution ~31 years) [33]. (**b**) March–November temperature, smoothed by a 10-year average, reconstructed from dO18 in ice cores of the Belukha glacier, Altai [28]. (**c**) Concentrations of HCOO^−^ (triangles) and NH_4_^+^ ions (circles) in the ice core at the same glacier [29]. Lines with markers represent temperature reconstruction or natural archive; line without markers represents PISIreg smoothed by 10-year (**a**,**b**) or 31-year (**c**) moving average.

However, not all proxy data support an extreme cold event ca 1700 CE in the study area (Figure 4). Pollen in the Hulun Lake sediments [33] recorded cold July temperatures in Mongolia for a century before that, but no sharp drop matching the nosedive. The 10-year-smoothed March–November temperatures in the Altai, reconstructed from the d18O in the Belukha glacier ice cores [28], had only a moderate negative deviation in 1691–1720 and a stronger one in 1741–1750. Of more than 90 reconstructions assembled by Christiansen and Ljungqvist [1] to describe variation in Northern Hemisphere temperature, six temperature reconstructions are in our study area [13,14,28,32,34,35], and only one of them [35] indicates moderate cooling ca 1700 CE.

### 3.2. Extenuating Circumstances

From the evidence just given, we conclude that warm season cold spells, as in 1937 and 1988, probably contributed to the low in *P. sibirica* growth ca 1700 CE. The remarkable occurrence of six consecutive years of PISIreg is more than three standard deviations below the mean; however, this likely requires additional environmental impact, such as damage to *P. sibirica* trees from weather or another factor. We dismiss earthquakes as a likely suspect, since even the strongest known earthquakes in Altai did not exhibit comparable consequences. Drought and related causes, including wildfires [4,36,37,38] can also be dismissed because the habitat of the most affected trees is moist and cool [27,39,40]. Defoliation of trees by insects could severely suppress growth [4,6], and the study area is home to the gypsy moth and Siberian silk moth [41]. The most growth-suppressed trees in our study, however, are outside the distribution range of those insects due to short cool summers and frosty winters and would be more so during a cold period [4,42].

Stress from extreme weather events is on the other hand a likely factor in the nosedive. Mongolian historical records mention cold-related events at the time over vast areas [43]: multiple winter dust storms and snowstorms were registered in 1695, 1697 and 1705, and “dzuds” were reported in 1696 and 1704. The term “dzud” embraces several winter weather extremes—including heavy snowfalls, severe frosts, and thaws followed by the formation of thick ice crust—leading to a lack of forage and die-off of livestock and thus important for Mongolian nomadic herders [44]. Some of these phenomena (heavy snowfall, thaw–freeze cycles), as well as snowstorms, indicate the establishment of winter cyclones or atmospheric fronts during 1695–1705, in contrast to the typical Siberian High anticyclone [13,45].

Snowstorms and strong winds can adversely affect forests through crown damage and defoliation [6,9,23,46]. Extreme freeze–thaw cycles could also lead to stress in evergreen conifers through physiological drought and desiccation of needles [47]. The strengthening of the nosedive with elevation is explained by the greater amount of snow and stronger winds. The observed more severe and prolonged nosedive in pine than in larch (Figure 2) could be explained by ecophysiological factors. The average 5–8 years retention of needles by *P. sibirica* [48] would slow down recovery after defoliation. The large crown and dense bunches of needles in *P. sibirica* could also present a better target for wind and for accumulation of ice and snow in the crown, increasing the risk of mechanical damage.

### 3.3. Accomplice from the Tropics

Although our literature search revealed no local volcanic eruptions in the mountains of south-central Asia at the time of the nosedive, a large tropical eruption ca 1695 CE has been implicated in catastrophic summer cooling ca 1700 CE in Scotland and most of Northern Europe [5,8,49,50]. The resemblance of the warm season composite 1938/1988 500 mb anomaly pattern (Figure 3) to the positive mode of the Scandinavia pattern suggests possible influence from the 1695 volcano on the *P. sibirica* growth in the nosedive through amplification of the positive-phase Scandinavia pattern. The cool-season linkage of the Scandinavia pattern to the 1938/1988 composite anomaly maps of 500 mb height and 2 m air temperature is less clear. We can speculate that winter circulation may have been disrupted after the 1695 volcano to favor high winds, heavy snow, and tree damage through pathways already mentioned. Such damage would exacerbate growth suppression in *P. sibirica* beyond that due to energy shortage in the unusually cool, wet summers.

## 4. Materials and Methods

### 4.1. Data Sources

To analyze the geographical distribution of the ca 1700 CE nosedive in conifer tree-ring width (TRW), a study area (40–60° N, 60–120° E) was defined that extends roughly 10° to the south and north and 30° to the west and east of the central Western Sayan Mountains, where the phenomenon was initially discovered (Figure 1a).

The authors’ own dendrochronological data, starting no later than 1690, include TRW measurements of Siberian stone pine (*Pinus sibirica*) at 7 sites in the Western Sayan, and Siberian larch (*Larix sibirica*) at one site within the above-mentioned region. Collection and processing of samples and dendrochronological measurements, chronology development, and dendroclimatic analysis for most of the chronologies have been published elsewhere [11,51].

This study also uses a TRW dataset of *Pinus sibirica* and *Larix sibirica* species publicly available in The International Tree-Ring Data Bank (ITRDB; https://www.ncei.noaa.gov/products/paleoclimatology/tree-ring, accessed on 11 September 2024), selected by geographic screening for location within 40–60° N 60–120° E and start year no later than 1690. Screening yielded 60 sampled sites of *Larix sibirica* and 7 sites of *Pinus sibirica*. (One of the *Larix* chronologies is listed in the ITRDB as *Larix gmelinii* Rupr., but the site is in the distribution area of *Larix sibirica*. The complete TRW dataset broadly covers the Altai–Sayan Mountain complex and the Mongolian Plateau. All TRW chronologies used in the study are “standard” (autocorrelation not modeled out) chronologies with ring-width trends removed by conservative approaches to avoid the removal of low-frequency climate signals [24].

The ITRDB, with the same geographic and time-coverage screening as above and selection of the “climate reconstructions” category, was also the source of time series representing temperature variation in the study area. Reconstructions with varying temporal resolution and intra-seasonal frames available from the ITRDB were used and analyzed. This selection also provided us with ion concentration data in ice cores from the Belukha glacier [29]. In addition, other published temperature reconstructions were used qualitatively, where publications (references in the main text) include temporal plots.

The list of Holocene volcanoes, their coordinates and information on eruptions were taken from the database ‘Volcanoes of the World’ (https://volcano.si.edu, accessed on 7 October 2024). Documentary historical evidence for the study area was taken from generalized sources for the territories of Russia and Mongolia on social and natural phenomena [43,52] and on earthquakes (NGDC/WDS Global Significant Earthquake Database https://www.ngdc.noaa.gov/hazel/view/hazards/earthquake/search, accessed on 10 October 2024).

Maps of anomaly fields of geopotential height (500 mb) and temperature (2 m) for 20th to 21st century years with lowest PISIreg were generated using a web-based NOAA tool for mapping “20th Century Reanalysis Monthly Composites” (https://psl.noaa.gov/cgi-bin/data/composites/plot20thc.v2.pl, accessed on 14 October 2024). From the online menu, we specified “20CRV3” as the data source and used the menu options to tailor the map domain. Information on Northern Hemisphere Teleconnections, including maps with centers of action and associated patterns of climate anomalies for the “Scandinavia” pattern, were accessed at the NOAA Climate Prediction website (https://www.cpc.ncep.noaa.gov/data/teledoc/teleintro.shtml, accessed on 7 October 2024).

### 4.2. Data Processing and Analysis

For all raw TRW measurements, standardization was performed in the ARSTAN program [53] by smoothing with a 67% cubic spline, then averaging all the series within the site by bi-weighted mean. To identify the presence and intensity of the growth decline, tree-ring chronologies were transformed into Z-scores (mean = 0; SD = 1), since chronologies have different ranges of variability.

For the chronologies of *Pinus sibirica* with a visible decline in growth around 1700 (Figure A1), a standard series of individual trees covering the period from 1690 were analyzed using the superimposed epoch method, revealing the period of the most pronounced suppression as 1699–1706 (Z-scores less than −1, including the range ± SE, standard error). The presence of a long-term (or any) suppression in growth in the chronologies under consideration was assessed by mean (minimum) values of Z-scores of local standard chronologies for 1699–1706, also comparing them with the threshold of −1 (i.e., a drop in growth by SD or more relative to the long-term average). For *Larix sibirica* chronologies with suppressed growth, a series of individual trees were also investigated using the superimposed epoch method to estimate years of suppression for this species.

The similarity between tree-ring chronologies among themselves and with other time series was estimated using paired Pearson correlation coefficients, either for the entire period of overlapping series or for the century-long period 1650–1750 including the phenomenon of interest. In addition, an 11-year smoothing filter (moving average) was applied to a time series with an annual time resolution, dividing the series into low-frequency and (by subtracting the smoothed series from the original) high-frequency components, for which a correlation analysis was also performed. Cross-correlation analysis was performed to find if there is a lag of tree-ring chronologies relative to other time series, separately for low- and high-frequency components.

For the pollen-based temperature reconstruction, having an uneven temporal distribution of data points, or nonuniform time step, the average temporal resolution was estimated by dividing the coverage period by a number of intervals between data points and rounding to an integer number of years. For comparison of the TRW chronology PISIreg (annual resolution) with lower-resolution time series, we smoothed chronology with a moving average of appropriate window width.

The selection of 1938 and 1988 as years for mapping 500 mb height anomalies and 2 m temperature anomalies was made by (1) converting the 1524–2022 time series of PISIreg to Z-scores, using the mean and standard deviation for that full length, (2) sorting the Z-scores from smallest to largest, and (3) selecting the 20th century years with the lowest Z-scores. Years 1938 and 1988 are the only years with Z < −2.0 and are low outliers in that they are much lower than the next-lowest (Z = −1.6 in 1914).

Outside of specialized tools, data processing and statistical analysis were performed in Microsoft Excel 2007 (v12.0).

## 5. Conclusions

Case studies of extreme events in climate reconstruction are important because those events often draw the most interest and are highlighted in placing current and projected climate variation in a long-term context. Lacking instrumental observations, we present evidence from available natural and historical archives as proxies for environmental change and events. Such evidence is not irrefutable, but points to: (1) tree damage by winter weather extremes as the most likely cause of the extreme growth suppression of conifers near the upper treeline in the Sayan–Altai region at the start of the 18th century; (2) a tropical volcanic eruption contributed to the severity of the event; and (3) atmospheric teleconnections, primarily through an amplified Scandinavia pattern, is a global-scale transfer mechanism that helps explain the exacerbation of tree-growth suppression in our study area. We invite the international scientific community to prove or disprove our opinion.

## Figures and Tables

**Table 1:** Statistics summarizing the relationships between PISIreg and temperature reconstructions based on other tree-ring data.

Temperature Reconstruction				Z-Score Mean/min, 1699–1706	Correlations with PISIreg, 1650–1750/Total Overlap Period ^b^				
Publication	Used Proxy ^a^	Season	Location		Annual	Smoothed ^c^	Smoothed (Lagged) ^d^	Lag, Years	High-Pass ^e^
[19]	LASI TRW	June–August	Altai	−1.56/−2.68	**0.52/0.30**	**0.73/0.20**	**0.78/0.22**	3/2	**0.41/0.41**
[20]	LASI TRW	June–July	Mongolia	−1.49/−2.45	**0.49/0.27**	**0.70/0.20**	**0.71/0.21**	1/2	**0.35/0.33**
[15]	PCSH MXD	July–August	Tien-Shan	−1.34/−1.97	**0.35**/0.04	**0.40**/0.06	**0.82/0.23**	7/8	−0.13/−0.03
[16]	LASI BI	June–August	Mongolia	−0.82/−2.01	**0.25/0.27**	**0.23/0.21**	**0.30/0.23**	4/3	**0.25/0.28**

^a^ Tree species: LASI—*Larix sibirica*, PCSH—*Picea schrenkiana* Fisch. & C.A.Mey. Tree-ring parameters: TRW—tree-ring width; MXD—maximum wood density; BI—blue intensity (proxy for wood density). ^b^ Bold correlation coefficients are significant at *p* < 0.05. ^c^ Time series were smoothed by an 11-year moving average. ^d^ Maximum cross-correlation between smoothed time series, with PISIreg delayed by “lag” years after temperature reconstruction. Plots for 1650–1750 are presented in Figure A2. ^e^ High-pass filter applied to both time series by subtracting smoothed series from original.

**Table 2:** Comparison of growth departures in tree-ring width chronologies of pine and larch for 8-year periods after the investigated event and after the three strongest known earthquakes in the Altai mountains.

Type of Event ^b^	Calendar Date	Epicenter Coordinates		Z-Scores ^a^		
		Lat. N	Lon. E	Range of Means		Period of Calculation ^c^
				Larch	Pine	
Unknown	ca 1698–1699	~47–53°	~85–95°	−2.61…0.95	−3.39…0.53	1699–1706
Earthquake	9 December 1761	~50°	~90°	−0.69…2.03	−0.32…1.48	1762–1769
Earthquake	4 December 1957	45.5°	99.5°	−1.16…1.00	−1.72…1.22	1958–1965
Earthquake	27 September 2003	50.038°	87.813°	−0.15…0.77	−1.01…1.54	2004–2011

^a^ Z-scores represent a complete set of 14 pine and 61 larch chronologies in the study area. ^b^ Event, factor, or their combination that led to the nosedive in tree growth investigated in this study. ^c^ Since all three earthquakes occurred after growth season, tree growth could react to them only beginning in the next year.

**Figure A2:**
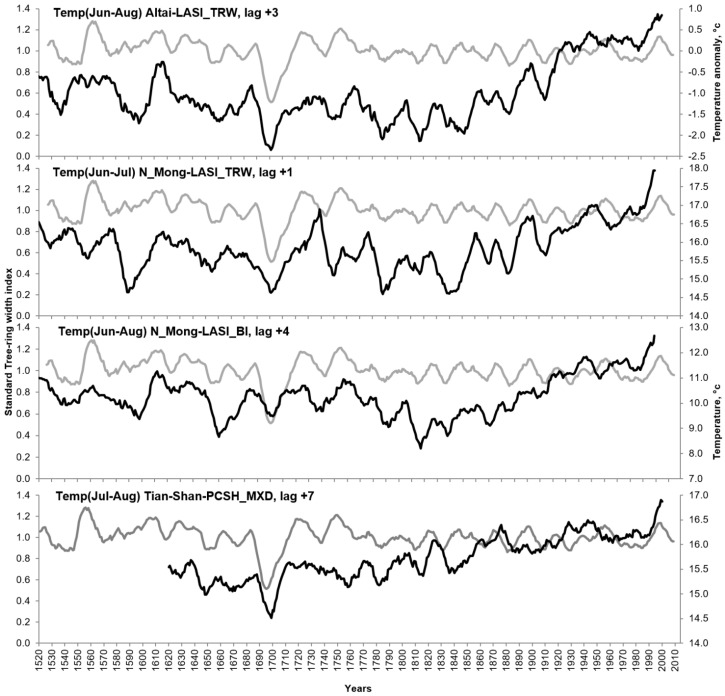
Cross-correlated decadal variations of *P. sibirica* growth and temperature reconstructions. Grey lines represent PISIreg chronology, lagged (shifted to the left) according to plot labels; black lines represent temperature reconstructions for analysis summarized in Table 1. Time series were smoothed by 11-year moving average. Plots truncated to start in 1520.

## Data Availability

Analyzed data from publicly archived datasets include tree-ring width measurements, climatic reconstructions and glacier ice composition data from the ITRDB (https://www.ncei.noaa.gov/products/paleoclimatology, accessed on 11 September 2024), data on volcanoes and their eruptions from the database ‘Volcanoes of the World’ (https://volcano.si.edu). data on earthquakes from the NGDC/WDS Global Significant Earthquake Database https://www.ngdc.noaa.gov/hazel/view/hazards/earthquake/search, accessed on 10 October 2024), and climatic data from the NOAA websites (https://psl.noaa.gov/cgi-bin/data/composites/plot20thc.v2.pl, accessed on 14 October 2024, and https://www.cpc.ncep.noaa.gov/data/teledoc/teleintro.shtml, accessed on 7 October 2024). Original data included in this study (tree-ring width measurements) will be available on request from the corresponding author.

## Appendix A

**Table A1:** List of used tree-ring width data sources.

No	Tree Species ^a^	Country	ITRDB Code or Chronology Code ^b^	Coordinates			Mean/min in 1699–1706 ^d^	*R* ^e^
				Lat. N	Long. E	Elev. ^c^, m a.s.l.		
1	LASI	Russia	RUSS127	50.15	85.37	1750	−0.47/**−1.64**	−0.03
2	LASI	Russia	RUSS129	51	85.63	1450	−0.31/**−1.43**	0.18
3	LASI	Russia	RUSS130	50.87	85.23	1450	−0.65/**−1.39**	**0.21**
4	LASI	Russia	RUSS135	50.42	87.58	2000	**−1.23/−2.66**	**0.48**
5	LASI	Russia	RUSS137	50.48	87.65	2150	**−1.20/−2.24**	**0.31**
6	LASI	Russia	RUSS226	50.803	87.933		**−2.61/−3.42**	**0.65**
7	LASI	Russia	RUSS227	49.617	88.1		**−2.09/−3.26**	**0.59**
8	LASI	Russia	RUSS228	50.867	85.233		−0.18/−0.93	0.14
9	LASI	Russia	RUSS229	50.3	87.833		−0.83/**−2.50**	**0.26**
10	LASI	Russia	RUSS230	50.267	87.833		−0.26/**−1.17**	0.08
11	LASI	Russia	RUSS231	49.167	87.283		−0.50/**−1.32**	0.04
12	LASI	Russia	RUSS232	50.167	87.833		**−1.86/−3.28**	**0.62**
13	LASI	Russia	RUSS233	50.117	87.917		−0.57/**−1.75**	**0.22**
14	LASI	Russia	RUSS234	50.483	87.483		**−1.27/−2.31**	**0.37**
15	LASI	Russia	RUSS235	50.683	87.967		**−1.12/−1.88**	0.19
16	LASI	Russia	RUSS241	52.399	98.685	2020	−0.15/−0.78	0.06
17	LASI	Russia	RUSS247	49.23	87.23	2200	**−1.19/−2.52**	**0.40**
18	LASI	Russia	RUSS248	49.2	87.02	2250	−0.70/**−1.70**	**0.31**
19	LASI	Russia	RUSS249	51.58	95.31	2060	−0.23/−0.64	0.18
20	LASI	Russia	RUSS250	49.51	87.5	2250	**−1.49/−3.45**	**0.50**
21	LASI	Russia	RUSS251	49.36	86.57	2200	−0.92/**−2.13**	**0.30**
22	LASI	Russia	RUSS252	50.36	91.28	2170	−0.66/**−2.68**	0.06
23	LASI	Russia	RUSS253	50.2	96.39	2254	−0.23/−1.00	**0.25**
24	LASI	Russia	RUSS254	50.24	89.59	2280	**−1.49/−2.94**	**0.52**
25	LASI	Russia	RUSS255	50.07	88.17	2100	−0.61/**−1.98**	0.17
26	LASI	Russia	RUSS256	50.49	94.18	2130	−0.31/**−1.13**	0.11
27	LASI	Russia	RUSS257	49.39	88.14	2250	**−1.16/−2.78**	**0.37**
28	LASI	Russia	RUSS258	50.22	98.14	2200	−0.62/**−1.12**	**0.35**
29	LASI	Russia	RUSS259	50.04	87.54	2250	**−1.37/−2.22**	**0.30**
30	LASI	Russia	RUSS288	56.412	115.589	465	−0.62/**−1.29**	**0.30**
31	LASI	Russia	RUSS292	53.144	106.832	810	**−1.03/−1.68**	0.18
32	LASI	Russia	AKK ^b^	49.917	86.55	2050	**−1.75/−3.09**	**0.55**
33	LASI	China	CHIN029	43.85	93.3	2810	0.11/**−1.54**	−0.04
34	LASI	China	CHIN030	43.833	93.383	2840	**−2.10/−3.41**	**0.49**
35	LASI	Mongolia	MONG001	50.77	100.2	2300	−0.09/−0.56	0.15
36	LASI	Mongolia	MONG006	47.78	107.5	1415	0.07/**−1.45**	−0.04
37	LASI	Mongolia	MONG009	49.92	91.57	2500	−0.26/−0.88	0.10
38	LASI	Mongolia	MONG010	47.27	100.03	2500	0.36/**−1.65**	−0.06
39	LASI	Mongolia	MONG011	48.15	100.28	1900	−0.30/**−1.38**	0.17
40	LASI	Mongolia	MONG012	48.98	103.23	1400	0.20/−0.94	0.00
41	LASI	Mongolia	MONG013	48.77	97.12	1840	**−1.14/−1.53**	**0.25**
42	LASI	Mongolia	MONG014	49.48	100.83	1800	−0.20/**−1.20**	0.10
43	LASI	Mongolia	MONG015	48.17	99.87	2060	0.14/**−1.13**	−0.02
44	LASI	Mongolia	MONG016	48.6	88.367		0.36/**−1.24**	**−0.24**
45	LASI	Mongolia	MONG017	49.967	91		**−1.06/−2.00**	**0.20**
46	LASI	Mongolia	MONG018	49.967	90.983		**−1.03/−1.92**	**0.33**
47	LASI	Mongolia	MONG019	47.1	90.967		0.85/0.03	−0.11
48	LASI	Mongolia	MONG020	48.267	88.867		0.45/−0.45	−0.13
49	LASI	Mongolia	MONG021	48.35	107.467		−0.28/−0.71	0.11
50	LASI	Mongolia	MONG023	49.5	94.583		−0.38/−0.92	0.02
51	LASI	Mongolia	MONG024	48.5	88.5		−0.07/**−1.22**	0.10
52	LASI	Mongolia	MONG025	48.7	88.8		−0.49/**−1.13**	0.06
53	LASI	Mongolia	MONG026	46.817	100.117		0.14/−0.87	0.01
54	LASI	Mongolia	MONG027	46.317	101.317		−0.67/**−1.81**	**0.30**
55	LASI	Mongolia	MONG028	48.833	111.683		0.95/−0.38	**−0.26**
56	LASI	Mongolia	MONG029	49.867	91.433		−0.76/**−1.72**	−0.03
57	LASI	Mongolia	MONG030	49.383	94.883		−0.52/**−2.27**	0.17
58	LASI	Mongolia	MONG032	46.517	100.95		−0.27/**−1.41**	**0.21**
59	LASI	Mongolia	MONG033	49.37	94.88	2229	−0.74/−0.99	**0.51**
60	LASI	Mongolia	MONG040	51.15	99.083	2400	**−1.29/−2.20**	**0.49**
61	LAGM	Mongolia	MONG007	49.7	91.55	2000	−0.44/**−1.90**	−0.10
62	PISI	Russia	RUSS222	50.417	84.617	1898	**−1.83/−2.54**	**0.64**
63	PISI	Russia	SPass20 ^b^	51.71	89.96	2000	**−3.39/−3.82**	**0.91**
64	PISI	Russia	GladSW ^b^	52.91	91.36	1620	**−3.17/−3.80**	**0.86**
65	PISI	Russia	SAR ^b^	52.23	92.25	1630	**−2.46/−3.27**	**0.85**
66	PISI	Russia	MGol8 ^b^	52.533	92.05	920	**−1.56/−2.20**	**0.49**
67	PISI	Russia	MGol5 ^b^	52.55	92.117	570	**−1.02/−2.01**	0.19
68	PISI	Russia	ErgV ^b^	52.8	93.433	1650	**−2.07/−3.99**	**0.75**
69	PISI	Russia	ErgR ^b^	52.833	93.333	1450	**−1.17/−1.87**	**0.57**
70	PISI	Mongolia	MONG041	48.17	99.87	2060	0.53/−0.67	0.00
71	PISI	Mongolia	MONG042	46.68	101.77	2125	0.42/−0.32	−0.12
72	PISI	Mongolia	MONG002	47.77	107	1755	**−1.45/−2.24**	0.13
73	PISI	Mongolia	MONG003	48.3	98.93	2420	−0.58/−0.93	**0.25**
74	PISI	Mongolia	MONG008	49.37	94.88	2229	**−1.66/−2.58**	**0.46**
75	PISI	Mongolia	MONG031	48.25	97.4		0.51/−0.72	−0.20

^a^ Species: LASI—*Larix sibirica* Ledeb.; LAGM—*Larix gmelinii* Rupr. (although, this is probably mislabeled LASI, considering species distribution areas); PISI—*Pinus sibirica* Du Tour. ^b^ Marked are chronologies collected by authors. Not marked are chronologies from The International Tree-Ring Data Bank (ITRDB; https://www.ncei.noaa.gov/products/paleoclimatology/tree-ring, accessed on 11 September 2024). ^c^ Elevation data are listed if they are mentioned in the ITRDB record or measured by authors. ^d^ Mean/minimal values are presented after transformation of chronologies into Z-scores. Bold values are those below −1 (i.e., below mean–SD in original standard chronologies), indicating long-term/any growth suppression. ^e^ *R* is correlation with PISIreg (generalized TRW chronology of *Pinus sibirica* from sampling sites SPass20, GladSW, SAR, ErgV) over 1650–1750. Bold values are significant at *p* < 0.05.
